# Supplementary material for: DnaE uses strand displacement synthesis during Okazaki fragment repair
Source: bioRxiv. 2026 Apr 9:2026.04.08.717263. Preprint. [Version 1] doi: 10.64898/2026.04.08.717263 (PMC13082043; doi:10.64898/2026.04.08.717263)
Supplement: Supplement 1 [file media-1.pdf]

Supplemental Information

DnaE uses strand displacement synthesis during Okazaki fragment repair

Abigail Kendal[1†], Frances Caroline Lowder[1†], Lina G. Jeffery[1], and Lyle A. Simmons[1*]

[1]Department of Molecular, Cellular, and Developmental Biology, University of Michigan, Ann Arbor, MI 48109.

[†]These authors contributed equally to this work.

*LAS: Department of Molecular, Cellular, and Developmental Biology, University of Michigan, Ann Arbor, Michigan 48109-1055, United States. Phone: (734) 763-7142, Fax: (734) 647-0884, E-mail: lasimm@umich.edu

Short title: DnaE functions during Okazaki fragment repair

Keywords: *Bacillus subtilis,* DNA replication, Okazaki fragments, FEN, RNase HIII

SUPPLEMENTAL INFORMATION

Table S1. Strains used in this study.

Strain	Background	Genotype	Source
AHK27	PY79	Spβ^0 prototroph	(1)
FCL15	PY79	*dinC-gfp* (spc^r)	(2)
FCL16	PY79	Δ*rnhC, dinC-gfp* (spc^r)	This work
FCL9	PY79	Δ*rnhC,* Δ*fenA, dinC-gfp* (spc^r)	This work
FCL4	PY79	Δ*fenA*	(3)
FCL11	PY79	Δ*rnhC*	(3)
FCL3	PY79	Δ*polA*	(3)
FCL5	PY79	Δ*fenA,* Δ*rnhC*	(3)
FCL12	PY79	Δ*rnhC, polA*	(3)
AHK85	PY79	*dnaE::dnaE-mCitrine*	JWS163 (lab stock)
AHK114	PY79	*dnaE::dnaE-mCitrine,* Δ*polA*	This work
AHK112	PY79	*dnaE::dnaE-mCitrine,* Δ*fenA*	This work
AHK113	PY79	*dnaE::dnaE-mCitrine,* Δ*rnhC*	This work
AHK125	PY79	*dnaE::dnaE-mCitrine, dnaX::dnaX-mCherry*	This work
AHK128	PY79	*dnaE::dnaE-mCitrine, dnaX::dnaX-mCherry,* Δ*polA*	This work

All strains are derivative of PY79.

Table S2. Oligonucleotides used in this study

Oligonucleotide	Purpose	Sequence (5′-3′)
oFCL6	Template	GCAATCGACTCGTAAGCATGGTTCACTACTAGCTGCACATCGCTGCTTGATGCTCAATCG
oFCL8	Primer	/5IRD800/C*G*A*TTGAGCATCAAGCAGCG
oFCL11	Ladder	/5IRD800/CGATTGAGCATCAAGCAGCGATGTGCAGCTAGTAGTGAACCATGCTTACGAGTCGA TTGC
oFCL28	Template	GCAATCGACTCGTAAGCAGTTGGACAGCAGAGCTGCACATCGCTGCTTGATGCTCAATCG
oFCL30	Downstream fragment	AGTAGTGAACCATGCTTACGAGTCGATTGC/3IR800CWN/
oFCL31	Primer	/5IRD700/C*G*ATTGAGCATCAAGCAGCG
oFCL32	Ladder	/5IRD700/CGATTGAGCATCAAGCAGCGATGTGCAGCTAGTAGTGAACCATGCTTACGAGTCGA TTGC
oJR361	Template	GCAATCGACTCGTAAGCATGGTTCACTACTCGCTGCTTGATGCTCAATCG
oJR362	Primer	/5IRD800/CGATTGAGCATCAAGCAGCG
oJR367	Downstream fragment	rArGrUrArGrUrGrArArCrCrATGCTTACGAGTCGATTGC/3IRD700CWN/

IRD indicates an infrared dye with the excitation noted (700 or 800 nM).
* indicates a phosphorothioate linkage. Ribonucleotides are denoted by a lowercase 'r'.

Table S3. Substrates used in this study

Substrate Type	Oligonucleotides	Ladder
Primed	oJR361, oJR362	oJR362, oFCL11
Nicked	oJR361, oJR362, oJR367	oJR362, oFCL11
10 nt gap	oFCL6, oFCL8, oJR367	oFCL8, oFCL11
10 nt gap with flap	oFCL28, oFCL8, oJR367	oFCL8, oFCL11
10 nt gap (DNA only)	oFCL6, oFCL31, oFCL30	oFCL31, oFCL32

Table S4. Plasmids used in this study

Plasmid Identifier	Vector	Insert
pFCL1	his-pE-SUMO	*fenA* (3)
pFCL3	his-pE-SUMO	*polA* (3)
pFCL22	his-pE-SUMO	*rnhC*

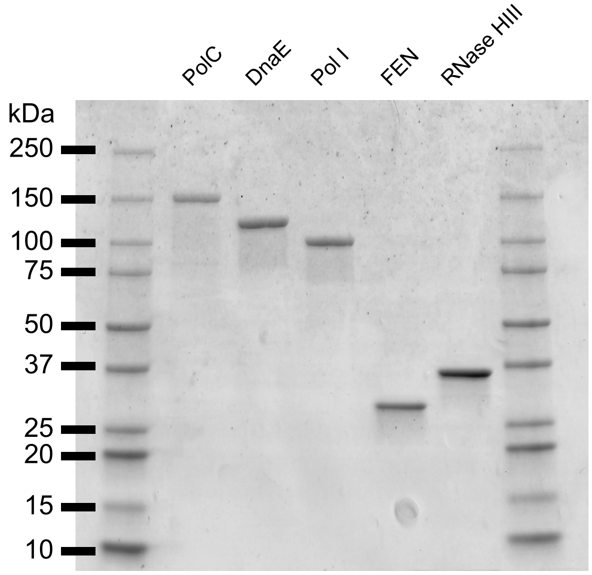

Figure S1. Proteins used in extension assays are clean purifications.
A total of 2 µg of each protein was separated following purification on SDS-PAGE as detailed in the Materials and Methods. SDS-PAGE was visualized following staining with Coomassie blue.

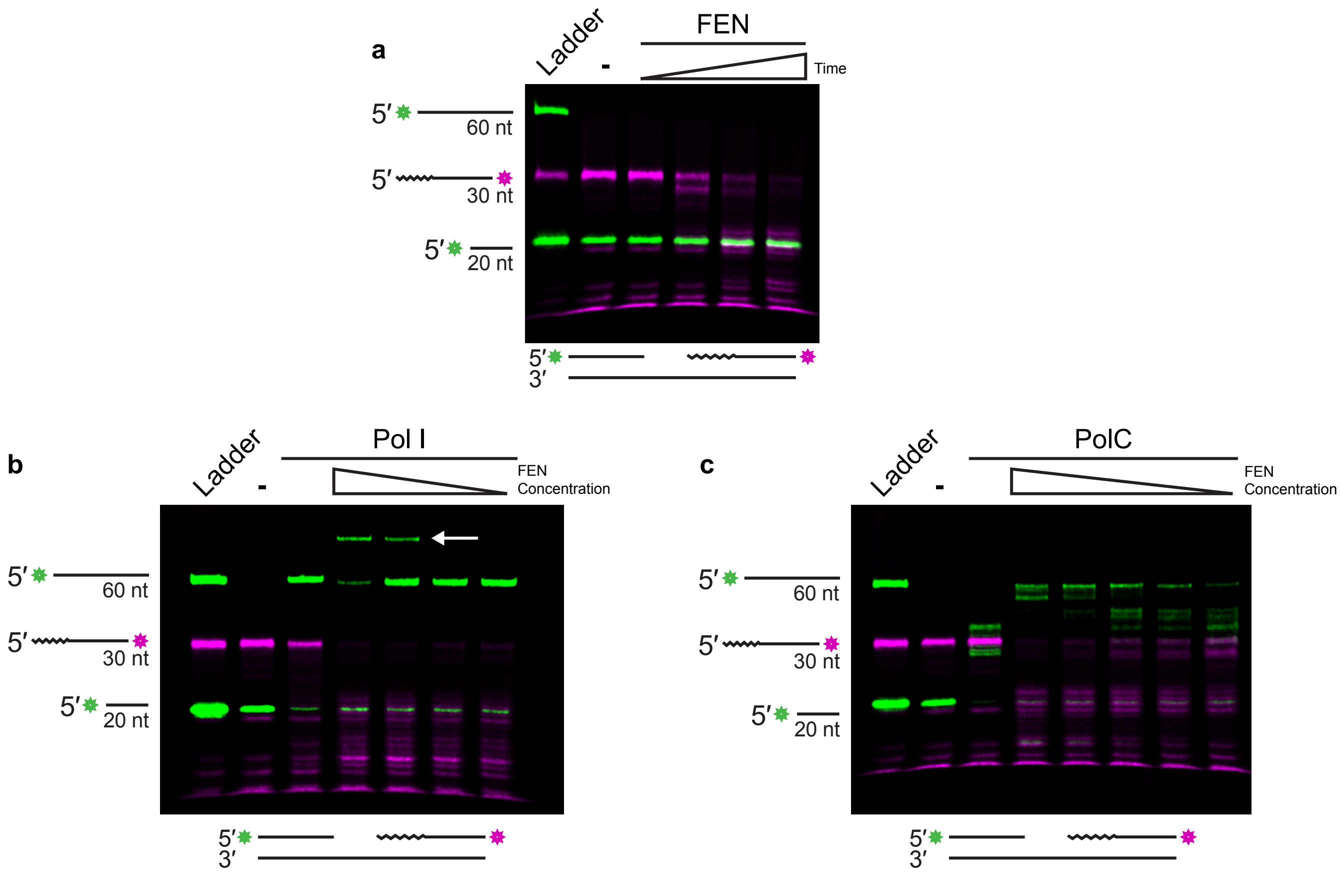

Figure S2. Relative degradation and extension products depend on protein concentrations.
(A) Products of an extension assay using a 10 nt gap substrate visualized by urea-PAGE. Ladder shows length of primer, downstream fragment, and full-length extension product. Embedded ribonucleotides denoted by squiggly line. Representative time increases from left to right with the following time points: 0 min, 1 min, 5 min, and 10 min.
(B) Products of an extension assay using a 10 nt gap substrate visualized by urea-PAGE. Ladder shows length of primer, downstream fragment, and full-length extension product. Embedded ribonucleotides denoted by squiggly line. FEN concentration decreases from left to right with the following concentrations: 50 nM, 25 nM, 10 nM, and 5 nM. Pol I concentration is consistent in all lanes (100 nM), except no protein control. Gel shift is denoted by white arrow.
(C) Products of an extension assay using a 10 nt gap substrate visualized by urea-PAGE. Ladder shows length of primer, downstream fragment, and full-length extension product. Embedded ribonucleotides denoted by squiggly line. FEN concentration decreases from left to right with the following concentrations: 50 nM, 25 nM, 15 nM, 10 nM, and 5 nM. PolC concentration is consistent in all lanes (100 nM), except the no protein control.

SUPPLEMENTAL REFERENCES

1. Youngman P, Perkins JB, Losick R. Construction of a cloning site near one end of Tn917 into which foreign DNA may be inserted without affecting transposition in Bacillus subtilis or expression of the transposon-borne erm gene. *Plasmid* 1984;12(1):1-
2. Britton RA, Kuster-Schock E, Auchtung TA, Grossman AD. SOS induction in a subpopulation of structural maintenance of chromosome (Smc) mutant cells in Bacillus subtilis. *J Bacteriol* 2007;189(12):4359-4366.
3. Lowder, F.C. and Simmons, L.A. (2023) Bacillus subtilis encodes a discrete flap endonuclease that cleaves RNA-DNA hybrids. *PLoS Genet*, 19, e1010585.
